# Trait-based cue Utilization and initial skill acquisition: implications for models of the progression to expertise

**DOI:** 10.3389/fpsyg.2014.00541

**Published:** 2014-06-03

**Authors:** Mark W. Wiggins, Sue Brouwers, Joel Davies, Thomas Loveday

**Affiliations:** Centre for Elite Performance, Expertise, and Training, Macquarie UniversityNorth Ryde, NSW, Australia

**Keywords:** skill acquisition, cue utilization, expertise, training

## Abstract

The primary aim of this study was to examine the role of cue utilization in the initial acquisition of psycho-motor skills. Two experiments were undertaken, the first of which examined the relationship between cue utilization typologies and levels of accuracy following four simulated, power-off landing trials in a light aircraft simulator. The results indicated that higher levels of cue utilization were associated with a greater level of landing accuracy following training exposure. In the second study, participants’ levels of cue utilization were assessed prior to two 15 min periods during which they practiced take-offs and landings using a simulated unmanned aerial vehicle (UAV). Consistent with Study 1, the outcomes of Study 2 revealed a statistically significant relationship among levels of cue utilization and the number of trials to criterion on the take-off task, and the proportion of successful trials during both take-off and landing. In combination, the results suggest that the capacity for the acquisition and the subsequent utilization of cues is an important predictor of skill acquisition, particularly during the initial stages of the process. The implications for theory and applied practice are discussed.

## INTRODUCTION

Expert performance across a range of environments, including sport, medical diagnosis, and financial decision making, is characterized by rapid, accurate responses, even in highly complex situations (Farrington-Darby and Wilson, 2006; Müller et al., 2006; Sherbino et al., 2012). Since this level of performance is generally acquired over extensive periods of exposure, there is an assumption that the capacity for sustained, high-levels of performance derives from the gradual development of highly specialized associations or routines that are subsequently retained in memory (Ericsson and Lehmann, 1996; Ericsson and Towne, 2010). Where these routines are available, they are activated rapidly and, in some cases, in the absence of conscious processing (Salthouse, 1991; Finkbeiner and Forster, 2007).

One of the advantages associated with the availability of highly specialized routines is that their activation imposes relatively fewer demands on working memory resources (Chung and Byrne, 2008). This enables experts to undertake multiple tasks simultaneously and with relatively consistent levels of accuracy (Houmourtzoglou et al., 1998; Boot et al., 2008). However, it also ensures that cognitive resources are available to enable both the acquisition of additional skills, and the refinement of those skills that have already been acquired.

The necessity for cognitive resources to facilitate the acquisition of cognitive skills reflects the theoretically important role of working memory in enabling the association between environmental features and objects or events. For example, in the case of production systems, Anderson et al. (2004) proposed that a production can only emerge when the condition and action statements are resident simultaneously in working memory. In the early stages of skill acquisition, the process of problem resolution involves the recall of declarative knowledge from long term into working memory, thereby occupying what is a finite resource. The development of a production or condition-action statement obviates this demand for declarative knowledge and the faster that this process occurs, the greater the capacity to allocate the residual resources to other tasks, thereby potentially improving the rate of skill acquisition.

The proposition that efficiencies in information processing can be gained through a parsimonious association between environmental features and events or objects is a consistent theme in various models of skill acquisition, as well as explanations of the superior performance of experts (Ericsson and Kintsch, 1995). Notions of bounded rationality, automated processing, and instance processing all presuppose tightly arranged associations to explain the rapid recognition and response to situations that are characteristic of expertise (Logan, 2002; Campitelli et al., 2007; Pachur and Marinello, 2013).

Klein (2011) suggests that the value of the associations in memory lies in their capacity to enable an operator to quickly classify or diagnose a situation. This process triggers an associated response from memory, and thereby facilitates a relatively rapid response. Where Anderson et al. (2004) would refer to this process as the activation of a condition–action statement in the form of a production, Gigerenzer and Gaissmaier (2011), Klein et al. (2010), and Brunswik (1955) suggest the application of cue-based associations. Cues constitute relationships among features and events or objects that are resident in the environment (Wiggins, 2006). They are highly specialized and targeted, and they enable the rapid recognition and response to particular situations.

The difficulty associated with the acquisition of associations between phenomena is that their coexistence does not necessarily infer a causal relationship (Holyoak and Cheng, 2011). A “storming” process is necessary during the acquisition of skilled performance whereby associations are quickly tested, discarded, or revised to ensure that they are both as accurate and as parsimonious as possible (Wiggins, 2012). However, an inevitable part of this process of storming is the commission of errors, whereby inappropriate cues may be triggered, or the cue associations themselves may be overly general or incorrect, thereby leading to inefficiencies or delays in the acquisition of skilled performance (Bridger and Mecklinger, 2014).

At a fundamental level, the capacity to acquire and subsequently revise cue-based relationships requires a cognitive strategy involving the identification of salient features in the environment, the perception of associations between features and events/objects, the retention of these associations in memory and, finally, the recognition of those situations during which the application of cues applies (Wiggins, 2014). The efficiency with which this process occurs will determine the rate of skill acquisition. However, what remains unclear is whether the capacity for the development and application of cues is determined by a particular context, or whether it constitutes an underlying trait so that the rate of cue acquisition within one context predicts performance in a related domain.

The aim of the present study was to consider the relationship between the cue utilization in the context of motor vehicle hazard detection and way finding, and skill acquisition in learning to land a simulated aircraft and learning to take-off and land a line-of-sight umanned aerial vehicle (UAV). If skilled psycho-motor performance is dependent upon the availability of feature–event/objects in memory in the form of cues, the rate at which cues are acquired within a given period should predict the rate of skill acquisition. Moreover, if cue acquisition is a trait, then the rate of cue utilization evident in one task should reflect the rate of skill acquisition in tasks that demand similar capabilities.

Although the relationship between cue acquisition and skill acquisition has yet to be examined empirically, some evidence for a relationship can be drawn from Small et al. (2014) who were investigating the relationship between cue utilization and performance during a novel, short vigilance task. Of particular interest in the context of the present study was the observation that participants’ performance differed in the rate at which they became familiar with a novel representation of a domain-related task. This difference in the rate of skill acquisition was not explained by years of industry-related experience, suggesting that the acquisition of cues may constitute an underlying trait.

## STUDY 1

Study 1 was designed to examine the relationship between a composite measure of cue utilization in the context of motor vehicle hazard and way-finding, and performance in learning to land a simulated aircraft as close as possible to a runway target. Since cue utilization represents the outcome of the process of cue acquisition, it was important to control for domain-related experience. To that extent, drivers’ years of experience were recorded, and these data were employed as a covariate to control statistically for exposure to the domain. It was hypothesized that, controlling for driving experience, participants who recorded greater levels of cue utilization would land closer to the runway target following four exposure trials.

### METHOD

#### Participants

A total of 51 university students (25 male and 26 female) were recruited for the study. These participants comprised first- and second-year psychology students who each received 0.5% course credit for their participation. Their ages ranged from 18 to 22 years (*M* = 20.27, SD = 1.601). The inclusion criteria comprised licensed drivers who had never previously flown a flight simulator.

#### Instruments

A demographic questionnaire required participants to indicate their age, sex, years of driving experience, weekly driving frequency, weekly frequency of video-game play, and their experience operating a flight simulator. Cue utilization was assessed using the EXPERT Intensive Skills Evaluation (EXPERTise; Wiggins et al., 2010) situation judgement test (SJT). Designed to measure performance on several cue-based processing and problem solving tasks, it provides a composite assessment of domain-related cue utilization.

EXPERTise incorporates experimental tasks that have separately and collectively been associated with differences in operational performance. They include a paired association task, designed to establish the availability of feature–event/object relationships in the form of cues, a feature identification task, designed to assess feature priming, and a feature discrimination task, designed to test the precision of cue-based associations in memory. Each task yields a distinct but complementary assessment of cue utilization that, in combination, provides an overall assessment of the utilization of cues in memory.

In the paired association task, participants were presented with two different terms (feature–event/object) that appeared adjacent to one another for 1800 ms. Using a six-point Likert scale, participants were asked to indicate the extent to which they considered the two words related. Examples included related terms such as “journey time” (event) with “car speed” (feature) and relatively less related terms such as “red traffic light” (feature) and “freeway” (object). Higher levels of cue utilization were associated with a greater variance in the perceived relatedness of terms (Ackerman and Rathburn, 1984; Morrison et al., 2013). The use of variance as a measure of cue utilization in this context is based on the assumption that, through experience, the associations between cues are better refined and thereby lead to a greater level of dichotomy in perceptions of the association between features and events/objects. The measure of performance has been used successfully to differentiate experts from non-experts in a range of context (Witteman et al., 2012)

For the feature identification task, participants were tasked with locating, as quickly as possible, a ball displayed at different locations within a static, complex driving scene; a process similar to measures of field dependence (Goodenough, 1976). In this case, lower response latencies reflect a greater capacity to extract key features from a complex array (Wiggins, 2014).

In the feature discrimination task, participants were provided with a hypothetical scenario (“After driving for some time, you become aware that you have lost your bearings... Surveying your surrounds, you see several cars driving with surfboards on their roofs. There are also several beachgoers and shoppers walking nearby…. In the distance, you can see high-rise apartment blocks as well as palm trees. There are also several street signs visible…”). After making a decision as to their most likely response under the circumstances, participants rated the importance of different features in the formulation of their response. In the driving version of EXPERTise, 14 features were presented during the feature discrimination task, to which participants responded using a 10-point Likert Scale, with higher ratings equating to a greater level of importance in the decision process. In this task, higher levels of cue utilization were reflected in higher variance in the ratings of the perceived relevance of features (Weiss and Shanteau, 2003; Witteman et al., 2012).

The construct validity of EXPERTise has been demonstrated in a number of different domains, whereby typologies that were formed on the basis of performance across the EXPERTise tasks differentiated both simulated and actual performance in the workplace (Loveday et al., 2013b, c; Loveday and Wiggins, 2014; Wiggins et al., 2014). The test–retest reliability of the typologies has been demonstrated in the context of power control operators at six monthly intervals, κ = *0.59* (Loveday et al., 2013a).

#### Flight simulator

A Redbird FMX-1000 flight simulator incorporating three degrees of freedom was used to position a simulated Cessna 172 at an altitude of 1000 feet and a distance of 1.5 km from the runway. Two large white bars positioned on the runway represented the target landing point. After landing, the aircraft was repositioned and this process was repeated for four trials.

#### Procedure

The participants completed the tasks individually, and all measures were presented sequentially in the same sitting. Having completed the demographic questionnaire, participants were directed to the on-line version of EXPERTise and they were asked to follow the instructions that were displayed on the computer screen. Once completed, participants entered the flight simulator and were briefed on the basic controls and the aim of the exercise. They then completed four trials, attempting to guide the aircraft to a landing on the runway.

### RESULTS

#### Cue utilization typologies

Prior to detailed analysis, it was necessary to identify the cue utilization typologies that corresponded to relatively higher and lower levels of cue utilization. These typologies were based on the outcomes of the EXPERTise tasks and were employed in this case due to the correspondence with previous methodological approaches to the application of EXPERTise-related outcomes (Loveday et al., 2013b, c; Loveday and Wiggins, 2014; Wiggins et al., 2014). The calculation of typologies began with the aggregation of the responses within the tasks, the calculation of *z*-scores, and a cluster analysis to identify whether two, meaningful typologies could be established. In the present study, two typologies were identified with centroids that corresponded to: (a) a lower response latency in the feature identification task, and higher variance in the paired association and feature discrimination tasks (higher cue utilization), and (b) a greater response latency in the feature identification task, and lower variance in the paired association and feature discrimination tasks (lower cue utilization). The cluster analysis classified 15 participants in the higher cue utilization typology and 33 participants in the lower cue utilization typology (see **Table 1**). The data for three participants were excluded due to missing data.

**Table 1:** Cluster centroids for the EXPERTise task scores for Study 1.

	Cluster 1 (*n* = 15)	Cluster 2 (*n* = 33)
Feature identification	–1.01	0.47
Feature discrimination	0.99	–0.45
Paired association	1.24	–0.54

#### Landing performance

Flight performance data comprised the primary dependent variable in the current study. Specifically, the flight task required participants to land the aircraft at a specific target located on the runway. The difference between a participant’s landing position (longitude and latitude) and the ideal landing location (longitude and latitude) formed the “flight performance” variable in the current study. This was measured in kilometers and calculated using a distance calculator for compass coordinates. Lower scores in flight performance represent a shorter distance from the landing target, and thus, greater accuracy during the flight task.

Five landing trials were conducted by each participant. The Shapiro–Wilks normality statistic for each landing trial was non-significant (*p* < 0.05) and inspection of the *P–P* plots indicated normal distributions. The landing trials were analyzed via repeated measures and *post hoc* pairwise comparisons. A significant main effect for landing performance, *F*(4,144) = 12.83, *p* = 0.000, suggested that landing accuracy differed over trials. *Post hoc* comparisons and inspection of means revealed a pattern of steady and statistically significant improvement in landing accuracy, until the final trial (fifth landing). Landing accuracy in the first trial (*M* = 0.66, *SD* = 0.18, *SE* = 0.030) significantly improved in the second (*M* = 0.58, *SD* = 0.19, *SE* = 0.032, *p* = 0.011), and performance in the second trial significantly improved in the third trial (*M* = 0.51, *SD* = 0.21, *SE* = 0.034, *p* = 0.000). Compared to the third trial, the fourth showed continued improvement, with a reduced mean distance from the target (*M* = 0.48, *SD* = 0.20, *SE* = 0.033). However, the fourth trial was not significantly different from the preceding trial (*p* = 1.00). The final trial indicated a *decrease* in performance and increased error (*M* = 0.49, *SD* = 0.26, *SE* = 0.043). Trial five was also not significantly different from trials two, three, or four. Taken together, this pattern suggests that learning occurred, and that performance in the final trial may have been affected by fatigue. For this reason, the landing performance for trial four was selected as the dependent variable in subsequent analyses, since it was at this point that optimal performance, following learning, had been achieved.

#### Cue utilization and landing performance

A univariate analysis of covariance (ANCOVA) was used to test the relationship between cue utilization and landing performance, and comprised the cue utilization typology as the independent variable (higher and lower), landing performance (as measured by the distance from the target on the fourth trial) as the dependent variable, and years of driving experience as a covariate. The results revealed a statistically significant main effect for cue utilization typology, *F*(1,45) = 4.18, *p* = 0.047. An inspection of the mean landing performance of the clusters indicates that participants with higher levels of cue utilization (controlling for driving experience) landed the aircraft closer to target (*Mean distance* = 0.37, *SD* = 0.23) than participants with lower levels of cue utilization (*Mean distance* = 0.53, *SD* = 0.19). This suggests that a relationship exists between cue acquisition and the acquisition of skilled performance in related, psycho-motor task.

Using hierarchical regression with change statistics, the variance in landing performance attributable to cluster was 11.5% and when driving experience was included in the model, the total proportion of the variance explained increased to 27%. This change represents an increase of 24%, and was statistically significant, *F*(1,45) = 9.49, *p* = 0.005. Partial correlations for driving experience and cluster revealed that the variance uniquely attributed to cluster was 8.5% and the variance uniquely attributed to driving experience was 17.4%.

## STUDY 2

Study 2 was designed to extend the outcomes of Study 1 by examining the relationship between driving-related cue acquisition and the development of skilled performance in the operation of a simulated UAV. If cue acquisition represents a precursor to skilled performance, then measures of cue utilization (controlling for driving experience) were expected to predict the number of trials required to reach a predetermined level of take-off and landing performance, together with the proportion of successful take-off and landing trials. Since the acquisition of cues is also likely to dependent upon the capacity to exclude extraneous information and thereby identify predictive feature–event/object associations, it was also anticipated that measures of sensory processing sensitivity (SPS) and attentional control would account for a proportion of the variance associated with the acquisition of skilled performance in operating the UAV. Higher levels of SPS and lower levels of attentional control are normally associated with clinical conditions and heightened arousal (Aron and Aron, 1997; Derryberry and Reed, 2002). Therefore, lower levels of SPS in combination with higher levels of attentional control and greater levels of cue acquisition were expected to account for a greater proportion of successful trials and trials to reach criterion than either variable in isolation.

### METHOD

#### Participants

A total of 50 university students participated in the study of whom 21 were male and 29 were female. They were recruited from the Psychology Research Pool, and each received 1% course credit for their participation. They were aged between 18 and 26 (*M* = 18.87, *SD* = 1.58), possessed a current motor vehicle driver’s license, and had no experience in remote control aircraft operation.

#### Instruments

As in Study 1, the participants completed a demographic questionnaire, including questions related to video game and driving experience, and then progressed to complete the on-line version of EXPERTise. They were subsequently asked to complete Aron and Aron’s (1997) Highly Sensitive Person Scale and Derryberry and Reed’s (2002) Attentional Control Scale. The 27-item Highly Sensitive Person Scale requires participants to indicate their response on a seven-point Likert scale. An example item is “Are you easily overwhelmed by things like bright lights, strong smells, coarse fabrics, or sirens close by?” Levels of sensitivity are calculated by summing the responses to the questions with higher scores associated with higher levels of SPS. The scale has adequate discriminant, convergent, and overall construct validity, and Cronbach’s alphas have been obtained in the range of 0.81–0.84, demonstrating adequate reliability (Jagiellowicz et al., 2010). An alpha of 0.77 was achieved in the present study.

The Attentional Control Scale is designed to measure an individual’s general capacity to focus attention on a task, to filter out distractions, shift attention between tasks, and to flexibly control thought (Derryberry and Reed, 2002). The 20-item scale required participants to indicate their response on a four-point Likert scale. An example item is “When concentrating, I can focus my attention so that I become unaware of what’s going on in the room around me?” Scores are calculated by summing the responses to the questions with higher scores associated with greater levels of Attentional Control. The scale has adequate discriminant, convergent, and overall construct validity in different populations (Fajkowska and Derryberry, 2010). A Cronbach’s alpha of 0.83 was achieved in the present study.

#### UAV simulator

Real Flight 6.0^TM^ was used to simulate the operation of a UAV. The simulator was displayed on a 40-inch monitor with control exercised using a standard remote control aircraft transmitter, incorporating two joysticks, one to control the pitch and roll of the aircraft and the other to control power.

For the take-off task, the UAV was located at the end of the runway, and participants were asked to accelerate the aircraft using the joystick and fly the aircraft down the extended center line of the runway through two virtual parallel lines. In the case of this computer program, the failure to position the aircraft within the parallel lines would result in the destruction of the aircraft, wherein the aircraft was repositioned at the take-off in preparation for the next trial. Similarly, in the case of the landing, the aircraft was positioned at altitude, a short distance from the runway along the extended center line. Participants were asked to advance or retard the throttle while maintaining directional control of the aircraft, and descend between two parallel lines. Similar to the take-off task, the failure to maintain the position of the aircraft within the two parallel lines would result in the destruction of the aircraft and a return to the landing approach position. The number of trials was recorded and criterion performance was set at three consecutive successful take-offs or landings to reduce the influence of accidental and sporadic successes.

#### Procedure

Once the initial questionnaires had been completed, participants were asked to stand in front of the UAV display and the task was described, together with instructions concerning the control of the simulated aircraft. Participants were advised that they were to try and ensure that the aircraft departed or landed within the parallel lines and that flight outside the parallel lines would result in the destruction of the aircraft with the requirement to restart the trial. Similarly, if the participant succeeded in completing a trial successfully, the aircraft trial would be restarted. Participants were advised that they would be given 15 min to complete as many successful trials as possible. The take-off trials always preceded the landing trials.

### RESULTS

The aim of Study 2 was to examine the differential effects of cue utilization, sensory processing sensitivity, and attentional control on trials to criterion in learning to operate a UAV simulator. Similar to Study 1, data arising from the EXPERTise SJT were aggregated and converted into *z* scores. However, unlike Study 1, typologies were not established. Instead, a grand mean was employed as an overall, standardized measure of cue utilization and to allow for regression analyses.

#### UAV performance

Four measures of UAV skill acquisition were used as dependant variables in the present study. The number of trials to achieve criterion performance (three consecutive successful trials) was used to establish the rate of skill acquisition. A descriptive analysis of the data revealed that skewness was outside normal limits. A square-root transformation was undertaken subsequently, which reduced the skewness to an acceptable level (<1).

The number of trials to reach criterion performance could not be used as a measure of the rate of skill acquisition, as only 31 of the 50 participants were able to complete three consecutive landings successfully. Consequently, a new categorical variable was calculated with two levels: those who were able to achieve landing criterion performance and those who were unable to achieve criterion performance.

The proportion of successful trials was included as a broader measure of skill acquisition that was influenced by both the rate of skill acquisition and the consistency of performance beyond the initial achievement of the criterion. Proportions were derived for both the take-off and landing tasks by calculating the number of successful trials as a proportion of the total number of trials completed by each participant. The mean number of total trials across all participants was 70.82 for the take-off task (*SD* = 10.312) and 76.46 for the landing task (*SD* = 11.38).

#### Modeling UAV performance

A measure of SPS was obtained by summing the responses to all 27 items of the Highly Sensitive Person Scale. The Attentional Control score was obtained by summing the responses for the reverse scored items (1, 2, 3, 6, 7, 8, 11, 12, 15, 16, and 20) from the Attentional Control Scale. A hierarchical multiple linear regression was used initially to determine the relationship among cue utilization scores, weekly videogame use, SPS, Attentional Control, driving experience, and the proportion of successful take-off trials. Entering the cue utilization score explained 19.7% of the variance in the proportion of take-off trials and was statistically significant, *F*(1,47) = 11.81, *p* < 0.01. This increased to 30.3% of the explained variance with the addition of Sensory Processing Sensitivity scores, a change that was statistically significant, *F*(1,46) = 7.15, *p* < 0.01. The addition of the remaining variables failed to increase the amount of variance explained, indicating that the proportion of successful take-off trials during skill acquisition of the UAV was best predicted by a combination of a higher level of cue utilization (β = 0.38) and lower level of sensory processing sensitivity (β = –0.33).

Consistent with the results associated with the proportion of successful take-off trials, levels of cue utilization and sensory processing sensitivity also provided the model of best fit for the proportion of successful landing trials using the UAV, *F*(2,47) = 8.33, *p* < 0.01. Specifically, 26.2% of the variance in the proportion of successful landing trials was predicted by higher levels of cue utilization (β = 0.37) and lower levels of sensory processing sensitivity (β = –0.29). Although this is slightly lower than the variance explained for take-off performance, it is of particular note that neither driving experience, videogame experience nor attentional control contributed significantly to the final model. As might be expected a strong correlation was evidence between take-off and landing performance using the UAV [*r*(50) = 0.69, *p* < 0.001].

In relation to the number of trials required to satisfy the take-off criterion, the regression model of best fit was restricted to the level of cue utilization, β = –0.41, *F*(1,42) = 8.58, *p* < 0.01, which explained 17% of the variance in performance. No other variables contributed significantly to the model, including sensory processing sensitivity. This suggests that, while sensory processing sensitivity may explain some of the variance associated with sustained performance beyond the achievement of a criterion level of performance, it is not predictive of the initial achievement of this criterion.

Since the nature of the data arising from the landing performance task precluded the use of linear regression, a logistic regression was employed in which the dependent variable comprised whether or not the participant achieved the landing criterion. Consistent with the data for the linear regression concerning the achievement of the take-off criterion, only cue utilization was retained as in the model of best fit, β = 1.68, *SE* = 0.584, Wald’s *X*^2^ = 8.27, Exp(*B*) = 5.35, *p* = 0.004. The results suggest that the odds of achieving landing criterion increased by a factor of 5.35 for each unit increased in the log concentration of cue utilization (Hosmer–Lemeshow *X*^2^ = 8.78, Cox and Snell *R*^2^ = 0.20).

## GENERAL DISCUSSION

The overall aim of this research was to examine the role of cue utilization in the early development of skilled performance in two related domains. Cue utilization has been established previously as a characteristic of expertise (Loveday et al., 2013c), and this suggests that the capacity to identify, acquire, and retain feature–event/object relationships in memory may be a necessary precursor for the acquisition of expertise. Understanding the role of cue acquisition at the initial stages of skill development potentially gives rise to a more complete model of the mechanisms that will facilitate the transition from novice through competence to expertise.

Study 1 sought to establish the relationship between cue utilization and performance in learning to land a simulated aircraft. Cue utilization was assessed using the driving-related version of EXPERTise, and this enabled the formation of typologies reflecting relatively higher or lower levels of cue utilization. The results revealed a statistically significant relationship between cue utilization typology and the proximity to the runway target following the fourth landing trial. Specifically, higher levels of cue utilization, controlling for driving exposure, were associated with a closer proximity to the landing target. This suggests an association between a measure of cue utilization and performance on a novel skill acquisition task.

To establish the generalizability of the outcomes of Study 1 and to explore additional explanations of the mechanisms of skill acquisition, Study 2 was designed to employ the same measure of cue utilization (driving-related) but considered the acquisition of skilled performance in the operation of a UAV. While conceptually similar to the operation of a flight simulator, the operator of a Line-of-Sight UAV controls the aircraft from the ground using a remote control device.

In addition to cue utilization, additional variables were incorporated into the analysis, including video-game use, SPS, and attentional control. It was surmised that, in combination with the capacity to associate feature–event/object relationships in the form of cues, the capacity to identify prospective features and events would depend upon the capacity to direct attention to particularly salient features and avoid being distracted by those features that are likely to embody little predictive capacity. The results revealed a strong model in which a combination of cue utilization scores and sensory processing sensitivity was most predictive of both the number of trials to reach criterion on the landing and take-off tasks, together with the proportion of successful trials. Specifically, greater levels of cue utilization and lower sensory processing sensitivity predicted 31.7% of the variance associated with the acquisition of take-off performance on the UAV simulator.

In combination, the results of the two studies suggest that cue utilization in one context may play a significant role in the initial acquisition of skilled performance in other, related tasks. The fact that cue utilization is also characteristic of expert performance suggests that cues may constitute a key cognitive mechanism by which skill acquisition occurs, even at the earliest stages of the process.

### THEORETICAL IMPLICATIONS

Although there have been a number of different theoretical propositions concerning the cognitive mechanisms that facilitate the progression to expertise, including cases, instances, and productions, the present study targeted behavior that was most closely associated with the utilization of cues. EXPERTise is designed to target a number of aspects of cue utilization, including the capability to discern key features from a complex visual background, the capacity to differentiate the strength of the relationship between different feature–event/object pairs, and the relative importance of features in the context of diagnosis.

On the basis of the relatively consistent relationship between the driving-related version of EXPERTise and performance on the skill acquisition tasks, it might be concluded that the capacity to identify features and discern the strength of relationships between features and events/objects, constitutes a capability that informs the acquisition of skilled performance on both a three-dimensional tracking task in the context of flight simulation, and take-offs and landings in the context of operating a UAV. Moreover, in cases involving the acquisition of novel skills, the rate of progression toward expertise may be dependent upon: (a) the extent to which key features can be identified; (b) their association with events/objects established and retained in memory; and (c) their accurate application during the process of skill acquisition.

Although the outcomes of present research do not necessarily discount the role of productions as an explanation of skill acquisition, given the conceptual similarities between the two constructs, the role of cases and instances as an explanation of the process is less clear. In particular, the predictive capacity of a process that deconstructs tasks into distinct feature–event/object relationships, coupled with the lack of domain-related knowledge on the part of participants gives rise to a cue-based explanation of psycho-motor skill acquisition, particularly at the initial stages of the process. This explanation is consistent with previous research establishing the role of cue-based training in developing the skills of novices in other domains (Abernethy, 1990; Wiggins and O’Hare, 2003; Markovits, 2013; Momm et al., 2013).

As a context-dependent measure of cue utilization, EXPERTise has differentiated performance among pediatricians, power controllers, and software engineers. It has also identified differences in the acquisition of skilled performance in the context of power control. In combination with the results of the present study, cue utilization, as measured by EXPERTise, appears to both differentiate the performance of different operators, and predict the rate and the achievement of skilled performance. However, despite the relative consistency of the outcomes achieved, a number of key questions remain that are relevant to those models of cognitive skill acquisition that posit that the progression to expertise is based on the acquisition and utilization of cues. First, and most important, EXPERTise is purported to measure cue utilization but, in the absence of neurological evidence, that argument will remain speculative. Second, longitudinal studies have yet to be completed that include competent practitioners. Much of the work thus far has focused on the performance of novices, the transition from competence to expertise, or on retrospective accounts of skill acquisition and experience from skilled performers (e.g., Young and Salmela, 2010). Little research has been undertaken that considers the key transition from novice to competence that, potentially, is the stage at which mental models are acquired and tested.

### APPLIED IMPLICATIONS

The practical implications of the present study include the identification of those practitioners who are relatively more likely to achieve criterion performance on a three-dimensional psycho-motor tracking task, together with the rate at which this progression is likely to occur. The associated benefits include a potential reduction in the costs of training and attrition due to the selection of candidates who are capable of acquiring particular skills more accurately and at a rate that reduces the investment in both time and access to expensive simulation technologies.

In addition to the selection of candidates, the results of the present study also enabled the development of interventions for those candidates who, having been selected, experience plateaus in their acquisition of skilled performance. The apparent key role of cue utilization during the initial stages of skill acquisition suggests that learning plateaus may be explained by the inability of the learner either to identify predictive features, and/or establish a relationship between predictive features and associated events or objects. Therefore, learning plateaus may be considerably shortened if learners can be directed toward those features that are most appropriate in the context of the problem being confronted. Evidence for the potential utility of this type of approach to initial learning can be drawn from Lagnado et al. (2006) who observed that learning environments that are directed toward the identification of feature-outcome relationships can facilitate the acquisition of cue-based associations that, in turn, lead to improvements in performance. Similarly, Wulf et al. (2000) observed that improvements in tennis could be achieved by directing learners’ attention toward what they referred to as the “antecedents” and “effects” of particular types of strokes from opponents. In identifying and remediating “gaps” in cue-based processing, it becomes possible to augment existing training initiatives, thereby maintaining an optimal rate of skill acquisition, irrespective of the nature of the learner.

## CONCLUSION

The aim of this paper was to establish whether a measure of cue utilization predicts the rate and the achievement of initial performance in the acquisition of two, three-dimensional tracking tasks. In Study 1, participants learnt to maneuver a simulated light aircraft to land nearest to a target on a runway. Study 2 involved learning to take-off and land a UAV using a remote control device. In both studies, a relationship was established between cue utilization and task-related performance, whereby relatively higher levels of cue utilization were associated with both a greater rate of skill acquisition and a greater proportion of successful trials in learning to operate the UAV. Given that cue utilization also differentiates greater from lesser performance among experienced operators, the results suggest that the acquisition and subsequent utilization of cues may play a significant role in facilitating the rate and the achievement of expertise.

## Conflict of Interest Statement

The authors declare that the research was conducted in the absence of any commercial or financial relationships that could be construed as a potential conflict of interest.
